# Nitrate supply and uptake in the Atlantic Arctic sea ice zone: seasonal cycle, mechanisms and drivers

**DOI:** 10.1098/rsta.2019.0361

**Published:** 2020-08-31

**Authors:** Sian F. Henley, Marie Porter, Laura Hobbs, Judith Braun, Robin Guillaume-Castel, Emily J. Venables, Estelle Dumont, Finlo Cottier

**Affiliations:** 1 School of GeoSciences, University of Edinburgh, James Hutton Road, Edinburgh EH9 3FE, UK; 2 Scottish Association for Marine Science, Oban, Argyll PA37 1QA, UK; 3 Department of Mathematics and Statistics, University of Strathclyde, Glasgow G1 1XH, UK; 4 Department of Arctic and Marine Biology, UiT The Arctic University of Norway, 9037 Tromsø, Norway

**Keywords:** nutrients, nitrate, sea ice, Arctic Ocean, Barents Sea, Atlantic water

## Abstract

Nutrient supply to the surface ocean is a key factor regulating primary production in the Arctic Ocean under current conditions and with ongoing warming and sea ice losses. Here we present seasonal nitrate concentration and hydrographic data from two oceanographic moorings on the northern Barents shelf between autumn 2017 and summer 2018. The eastern mooring was sea ice-covered to varying degrees during autumn, winter and spring, and was characterized by more Arctic-like oceanographic conditions, while the western mooring was ice-free year-round and showed a greater influence of Atlantic water masses. The seasonal cycle in nitrate dynamics was similar under ice-influenced and ice-free conditions, with biological nitrate uptake beginning near-synchronously in early May, but important differences between the moorings were observed. Nitrate supply to the surface ocean preceding and during the period of rapid drawdown was greater at the ice-free more Atlantic-like western mooring, and nitrate drawdown occurred more slowly over a longer period of time. This suggests that with ongoing sea ice losses and Atlantification, the expected shift from more Arctic-like ice-influenced conditions to more Atlantic-like ice-free conditions is likely to increase nutrient availability and the duration of seasonal drawdown in this Arctic shelf region. The extent to which this increased nutrient availability and longer drawdown periods will lead to increases in total nitrate uptake, and support the projected increases in primary production, will depend on changes in upper ocean stratification and their effect on light availability to phytoplankton as changes in climate and the physical environment proceed.

This article is part of the theme issue ‘The changing Arctic Ocean: consequences for biological communities, biogeochemical processes and ecosystem functioning'.

## 1. Introduction

### (a) Arctic sea ice changes and primary production

The Arctic is one of the fastest-warming regions on Earth, with atmospheric warming occurring at approximately double the global average rate [1], due to amplification of anthropogenic climate change by sea ice losses and strong albedo feedbacks on upper ocean heat uptake [2]. Rapid and large-scale declines in sea ice extent and volume in recent decades, driven by this warming trend [3–5], are extending the ice-free period across large parts of the Arctic Ocean and widening the seasonal ice zone as multi-year ice is replaced by first-year ice (e.g. [6,7]). The reduction in Arctic sea ice varies regionally [8], and the northern Barents Sea has experienced a dramatic 3.5-fold increase in open-water duration over the past 40 years [9] with reductions in sea ice extent in all seasons [8].

An overall increase in primary production has been documented over recent decades using satellite-based estimates, and ascribed to the widening seasonal ice zone and hence longer phytoplankton productive season over a larger area of the Arctic Ocean [9–13]. The largest increases in primary production have been observed in the interior shelves (Kara, Laptev, East Siberian and Beaufort Seas) and the inflow shelves (Barents, Bering and Chukchi Seas) [14,15].

Sea ice cover exerts a major control on ocean physics, biogeochemistry and structuring of Arctic marine ecosystems [16]. Primary production is regulated by seasonal variability in light (photosynthetically active radiation, PAR), nutrient availability and sea surface temperature, each of which is intricately linked to the seasonal sea ice cycle and its coupling with upper ocean physics [14,17–23]. Nutrients are supplied from subsurface water masses of Atlantic or Pacific origin to the euphotic zone by vertical mixing, as well as regional upwelling, with each of these processes being modulated by sea ice dynamics [24–26]. The phytoplankton spring bloom is initiated when PAR increases after the polar night, nutrient concentrations are high after autumn/winter replenishment, temperature increases and mixed layer depth decreases as surface waters are stratified by solar heating and/or sea ice meltwater input [27–32]. Nitrate is the primary limiting nutrient for new exportable production, reaching low concentrations after the spring bloom [21,22,24,33]. Nitrate availability thus exerts an important constraint on the extent to which primary production can increase in response to increasing light availability with ongoing sea ice declines [26,34–36]. In shelf regions, additional blooms in summer/autumn can contribute significantly to primary production, consisting of phytoplankton acclimated to warm stratified low-nutrient waters, or if nutrient resupply from underlying waters and/or remineralization of the initial bloom occurs when PAR remains sufficient to support growth [24,29,31,37–39].

Continued increases in total Arctic Ocean integrated primary production (7–23%) have been hypothesized in response to increasing light and temperature as summer sea ice cover declines to zero over the coming decades [13,40]. However, there is strong regional variability in observed and projected trends [13–15,21,22], and disagreement between models as to whether primary production will increase or decrease overall over the twenty-first century, with surface stratification and nutrient availability being key factors [41]. For example, in the Canadian High Arctic sea ice declines are increasing upper ocean stratification, leading to reductions in primary production due to restricted vertical nutrient supply, despite increases in growing season length [18,42]. By contrast, nutrient supply is expected to increase and support increases in primary production in less-stratified regions, particularly along the shelf breaks, in response to projected increases in frequency and intensity of episodic vertical mixing and upwelling as ice retreats northwards, storminess increases and the ice-free season extends [13,20,22,26,39,43–45]. The largest increases in primary production are projected along the Eurasian shelf break, particularly in the northern Barents and Kara Seas [22].

### (b) The Barents Sea

**Figure 1.**
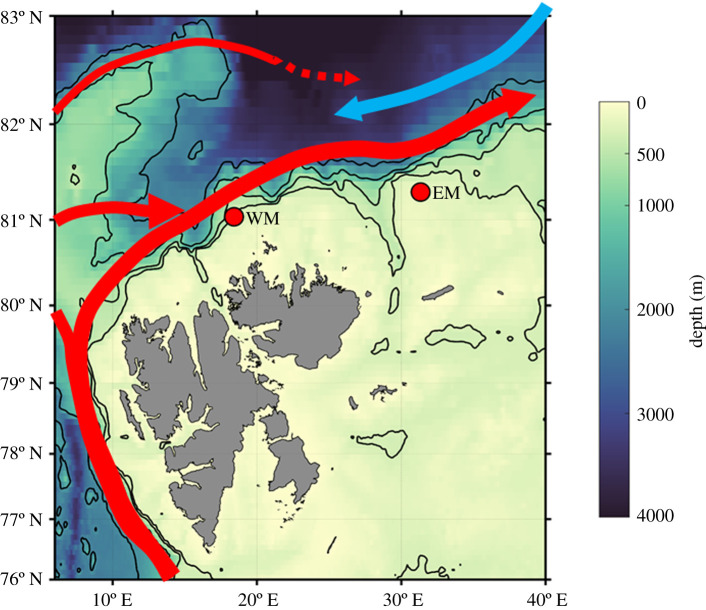
Map of the northern Barents Sea and shelf north of Svalbard, showing the positions of the Arctic PRIZE eastern and western moorings (EM and WM; table 1). Colour scale is depth with isobaths marked in black at 300 m, 500 m, 1000 m and 2000 m. Red arrows depict the position of the Atlantic Water boundary current; blue arrow depicts the easterly flow of Polar Surface Water.

The Barents Sea is an Atlantic inflow shelf, where the advection of nutrient-rich Atlantic-sourced waters provides the dominant nutrient source, and riverine inputs are small [14,36,46]. In our study region on the northern Barents shelf (figure 1), oceanography is dominated by three water masses; Atlantic Water (AW) advected along the northern slope from the west, Barents Sea Water (BSW) from the central Barents Sea to the south and Polar Surface Water (PSW) from the north and east [47,48]. Nutrient supply from AW and BSW to the overlying PSW occurs by autumn and winter mixing, upwelling along the shelf break and Polar Front, tidal forcing and episodic wind-mixing in spring/summer [31,37,49–51]. Recent evidence from the shelf-slope north and west of Svalbard shows that thermally induced convective mixing [52] is more important than wintertime shelf-break upwelling in the AW inflow region [53].

**Table 1.** Position, water depth and timings of deployment and recovery for the Arctic PRIZE eastern and western moorings.

	latitude	longitude	water depth	deployment date	recovery date
eastern mooring (EM)	81° 18.15	31° 20.57	182.5 m	21 September 2017	17 June 2018
western mooring (WM)	81° 02.03	18° 24.80	234 m	23 September 2017	14 June 2018

Atlantification—the increasing advection of warm (greater than 0°C), saline, nutrient-rich AW at intermediate depth (150–900 m)—is occurring along the continental slope north of Svalbard and over the Barents shelf from the south [24,54–57]. This northwards encroachment of mid-latitude-sourced waters increases heat and nutrient fluxes to overlying surface waters, accelerating the sea ice declines already underway [54,55,58] and potentially driving further increases in primary production [22,24,52].

### (c) Study objectives and outline

In order to better predict future changes in Arctic Ocean primary production, there is a pressing need to develop our mechanistic understanding of nitrate dynamics in different regions now and under climate change scenarios (e.g. [59]). Here we examine the seasonal changes in nitrate dynamics on the northern Barents shelf under contrasting sea ice conditions, with the aim of better constraining nitrate supply and uptake mechanisms and their physical and biological drivers over the annual cycle. We present seasonal time series (autumn to summer) of nitrate concentration and hydrographic data from two oceanographic moorings at very similar latitudes on the continental shelf north of Svalbard sited at similar water depths and distances from the shelf break. The similarity in latitude dictates that the timing of the return of the sun, day length at any time point and solar angle were matched for both moorings, negating the influence of these factors on the onset of phytoplankton growth [28,60]. The major difference between the mooring locations is that the eastern mooring was mostly ice-covered during autumn, winter and spring while the western mooring was ice-free year-round. This allows us to examine the influence of sea ice dynamics and coupled upper ocean processes on seasonal changes in nitrate supply and uptake and, by inference, primary production, independent of variability in solar forcing and water depth.

Improving our understanding of seasonal nutrient dynamics and drivers on the northern Barents shelf will contribute to modelling efforts focusing on ongoing and future changes in primary production in this biologically important and climatically sensitive shelf region as warming, Atlantification and ice losses continue. Changing nutrient dynamics and primary production have major implications for food web structure at all trophic levels [24,32,61] and for carbon export and biogeochemical cycling at the pan-Arctic scale [10,21,62].

## 2. Materials and methods

### (a) Moorings

Two oceanographic moorings were deployed on the northern Barents shelf (figure 1) from September 2017 until June 2018 (table 1). Each mooring was equipped with a SUNA V2 (submersible ultraviolet nitrate analyser) with biofouling wiper and an SBE16Plus conductivity-temperature-depth (CTD) unit with WETLabs ECO fluorescence and LICOR biospherical PAR sensors at a nominal depth of 21 m. Additional SBE37 CTD units and Star-Oddi temperature sensors were positioned at 4–25 m intervals over the full length of each mooring (to 170 m at the eastern mooring and 220 m at the western mooring), with higher resolution in the upper 100 m. A CTD profile was performed at each mooring site immediately prior to mooring deployment and recovery using the CTD packages on RV Lance (Seabird SBE911Plus unit including two WETLabs fluorometers) and RRS James Clark Ross (Seabird SBE911Plus unit including a LICOR PAR sensor and Chelsea Aquatrakka III fluorometer) for comparison with hydrographic instrumentation on the moorings.

SBE16 and SBE37 data were processed in Seabird software, where pre-deployment laboratory calibrations were applied for temperature, salinity and pressure, and additionally for PAR and fluorescence for SBE16 data. SBE16 and SBE37 data were handled further in Matlab and compared to deployment and recovery CTD profiles to check for offset and drift. In situ temperature and practical salinity were converted to conservative temperature and absolute salinity [63], which are presented and discussed throughout this paper. Potential density (*σ_θ_*) was calculated from conservative temperature and absolute salinity according to McDougall and Barker [63].

The percentage contributions of each of the three dominant water masses of the northern Barents shelf (AW, BSW, PSW) were calculated from mooring temperature and salinity measurements using a method of mixing triangles [64] employing single-point temperature and salinity definitions adapted from Oziel *et al.* [47] (table 2). We only show the dominant water mass at each time and depth, and thus percentage contributions of each water mass between 50 and 100% are used to track water mass variability with depth and over time at each mooring.

**Table 2.** The single point water mass definitions based on conservative temperature and absolute salinity used to calculate water mass percentage contributions to individual water samples [47].

water mass	temperature (°C)	salinity (g kg^−1^)
Atlantic Water (AW)	4	35.15
Barents Sea Water (BSW)	0	34.90
Polar Surface Water (PSW)	−1	33.15

Stratification is quantified as an index using the calculated potential energy anomaly [65], which is the potential energy (per m^2^) relative to a mixed water column. Greater values of stratification index indicate a more stable water column. Mixed layer depth was calculated as the depth of the maximum buoyancy frequency (N^2^), and showed that both SUNAs and the uppermost CTD packages were within the surface mixed layer throughout the deployments (electronic supplementary material, figure S1). Furthermore, mixed layer depths calculated from CTD calibration profiles were also deeper than the uppermost instruments (21 m); 25 m on deployment and 26 m on recovery at the eastern mooring, 75 m on deployment and 58 m on recovery at the western mooring.

SUNA nitrate sensors sampled absorption between 217 and 240 nm along a 10 mm path length, with a 30-s sampling interval of 28 light frames and 1 dark frame every 2 h. On recovery, SUNA calibrations were checked by measurements of known concentrations of potassium nitrate standard solution and reference spectra were updated using ultrapure deionized water. Nutrient samples were collected from each calibration CTD profile during mooring recovery, using niskin bottles attached to the CTD rosette to sample over the full water column depth, with higher vertical resolution around the depth of the SUNAs. Nutrient concentrations were analysed onboard using a Lachat Quikchem 8500 flow injection analyser standardized using international certified reference materials for nutrients in seawater (KANSO Ltd. Japan). Analytical precision is ± 0.2 µmol l^−1^ and the detection limit for nitrate is less than or equal to 0.35 µmol l^−1^.

SUNA data were processed according to Sakamoto *et al.* [66] using their temperature-compensated, salinity-subtracted algorithm in SeaBird UCI software version 2.0.3 and corrected for baseline drift using the reference spectra measured on recovery. Temperature and salinity data used in these corrections came from the paired SBE16 instrument for the western mooring and an SBE37 instrument immediately below the SUNA frame for the eastern mooring because the paired SBE16 failed. Processed nitrate data were compared to mean nitrate concentrations measured in seawater samples over the depth range between 7 m above and 7 m below the SUNA depth at each mooring location. These depth-based comparisons are robust because mooring-derived seawater density was equal to CTD-derived density at 21–23 m, and using the stated depth range gives a realistic estimate of uncertainty due to vertical displacement of water and associated gradients. These comparisons indicated an overall uncertainty in absolute nitrate values of approximately 1 µmol l^−1^ associated with the SUNA data acquisition and processing routines. This represents a large relative uncertainty at low nitrate concentrations and a need for caution when interpreting nitrate values below 1 µmol l^−1^. Standard deviation for each sampling interval for the eastern mooring SUNA was generally better than 0.2 µmol l^−1^ with a mean standard deviation of 0.11 µmol l^−1^, such that the minimum detectable difference in concentration was approximately 0.33 µmol l^−1^ (3 × SD [67]). For the western mooring SUNA, standard deviation for each sampling interval was generally less than 0.15 µmol l^−1^, mean standard deviation was 0.08 µmol l^−1^, making the minimum detectable concentration difference 0.24 µmol l^−1^.

### (b) Ice

Sea ice concentration (%) was determined using daily 3.125 km resolution Advanced Microwave Scanning Radiometer 2 (AMSR2) data downloaded from https://seaice.uni-bremen.de/sea-ice-concentration/ [68]. The pixel closest to each mooring (0.58 km from the eastern mooring, 0.39 km from the western mooring) was used to extract time series of concentration.

### (c) Wind

Wind stress was calculated using 10 m wind vectors extracted from the ERA5 reanalysis dataset [69] and sea ice concentration from AMSR2 data, following [70]. This representation of wind stress reflects the impact of the wind on the ocean surface and its modulation by sea ice cover.

## 3. Results

### (a) Seasonal variability in sea ice cover at the eastern and western moorings

**Figure 2.**
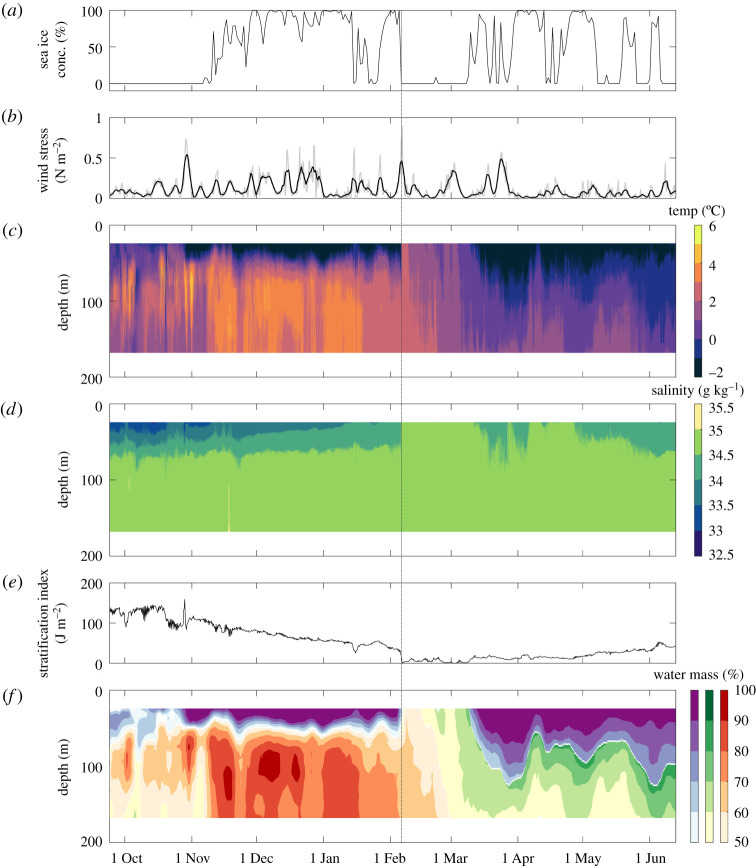
Time-series data for the eastern mooring, September 2017 to June 2018. (*a*) Sea ice concentration, (*b*) wind stress (4 hourly data in grey and a running 24-h mean in black), (*c*) conservative temperature, (*d*) absolute salinity, (*e*) stratification index and (*f*) water mass percentages (Polar Surface Water (PSW) = purple, Barents Sea Water (BSW) = green, Atlantic Water (AW) = red). The dashed vertical line is included to emphasize the co-occurrence of the wind stress peak in early February with the removal of ice cover and shift in water mass structure.

**Figure 3.**
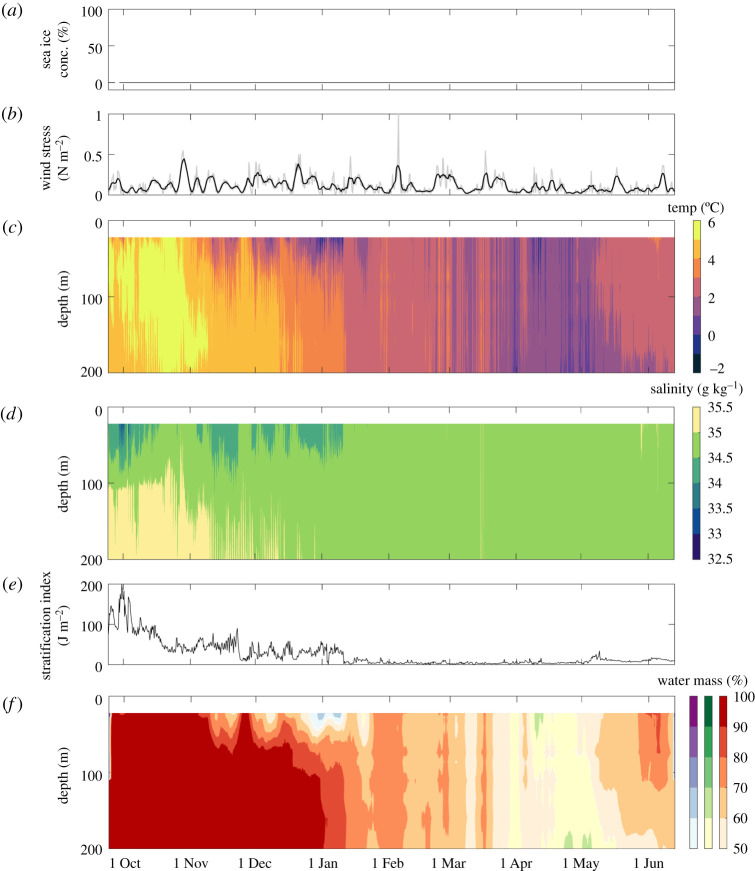
Time-series data for the western mooring, September 2017 to June 2018. (*a*) Sea ice concentration, (*b*) wind stress (4 hourly data in grey and a running 24-h mean in black), (*c*) conservative temperature, (*d*) absolute salinity, (*e*) stratification index and (*f*) water mass percentages (Polar Surface Water (PSW) = purple, Barents Sea Water (BSW) = green, Atlantic Water (AW) = red).

Sea ice was absent at the eastern mooring until early November (figure 2*a*). Sea ice concentration was then variable, but showed an overall increase to 100% by the end of November. A continuous ice cover of 100% concentration is rare in this dynamic and oceanographically complex region, but cover was mostly greater than 75% throughout December and early January. Ice cover was highly variable (0–75%) in mid-late January before reaching 100% again in early February. From 6 February to 8 March, the eastern mooring was almost completely free of sea ice. Ice concentration was then highly variable (ranging from 0 to 100%) to early June, with two sustained periods of high ice cover (greater than 90% concentration) in early April and late April to early May. The mooring was again almost completely free of sea ice from 8 to 18 May. Ice concentration was then variable until the eastern mooring became fully ice-free from early June until mooring recovery. By contrast, sea ice was totally absent at the western mooring throughout the study period (figure 3*a*).

### (b) Seasonal variability in water column structure, properties and wind forcing at the eastern mooring

Time-series fields of temperature, salinity and derived water mass percentages over the upper 170 m at the eastern mooring are shown in figure 2 with time series of stratification index and wind stress. Wind stress was highly variable over the study with a number of short-lived larger peaks superimposed onto a baseline of 0–0.20 N m^−2^. The largest wind stress peak (greater than 0.80 N m^−2^) occurred in early February 2018. Temperature and salinity showed the expected structure for the marginal ice zone of this Atlantic inflow shelf with warmer more saline Atlantic-sourced waters underlying cooler fresher PSW. Stratification index was highest in autumn 2017 and decreased to a minimum in early February. The rate of stratification breakdown between mid-October and early February was 1.3 ± 0.2 J m^−2 ^d^−1^. Stratification increased slightly from early March, but remained much lower than the autumn maximum until mooring recovery in June 2018. The water mass percentages derived from temperature and salinity show that the water column was dominated (greater than 50%) until early February by AW below approximately 50 m and by PSW in the upper 50 m; the two layers being separated by a distinct thermocline and halocline. In early February, a significant shift occurred whereby the two-layer structure collapsed and relatively uniform water properties extended over the full depth of the mooring array. PSW was not present down to 21 m (the depth at which the SUNA was moored), and was replaced by AW or BSW. The shift occurred immediately after the wind stress peak in early February and coincided with the prolonged period of open-water conditions from early February until early March. When the sea ice cover was re-established in early March, the upper water column was again occupied by PSW until mooring recovery. The layer thickness of PSW varied from less than 21 m in early March to 60–120 m as the pycnocline deepened in late March and again in late May and June. Beneath this PSW, the more mixed AW was replaced by BSW in late February, which persisted to at least 170 m for the remainder of the study.

### (c) Seasonal variability in water column structure, properties and wind forcing at the western mooring

Time-series fields of temperature, salinity and derived water mass percentages over the upper 200 m at the western mooring are shown in figure 3, with time series of stratification index and wind stress. The wind stress showed similar patterns to those at the eastern mooring, with larger peaks superimposed onto a baseline of 0–0.20 N m^−2^, but the magnitude of peak wind stress was lower overall (less than 0.5 N m^−2^). Nevertheless, the duration of these wind stress peaks was longer at the western mooring, such that wind forcing of the upper ocean may have been more sustained albeit less intensely than at the eastern mooring. This is consistent with the lack of ice cover throughout the study. Temperature and salinity were both substantially higher than at the eastern mooring and showed much more vertical homogeneity and less stratified water overall. This is consistent with a rapid reduction in stratification from the autumn maximum through October at a rate of 5.7 ± 0.7 J m^−2 ^d^−1^, a continued reduction overall into January and very low values for the remainder of the study. Water mass percentages showed less structure with depth than at the eastern mooring, with AW dominating the water column between 21 m and 200 m for the majority of the study. PSW occupied the upper water column down to approximately 40 m in late December and early January, and either shoaled above 21 m or was displaced by deeper waters to the surface for the rest of the deployment. BSW contributes more than 50% over the full 21–200 m depth range in April and May, until AW takes over again as the dominant water mass over the upper 200 m until mooring recovery.

### (d) Seasonal variability in upper ocean nitrate concentration, temperature, salinity and density at the eastern mooring

**Figure 4.**
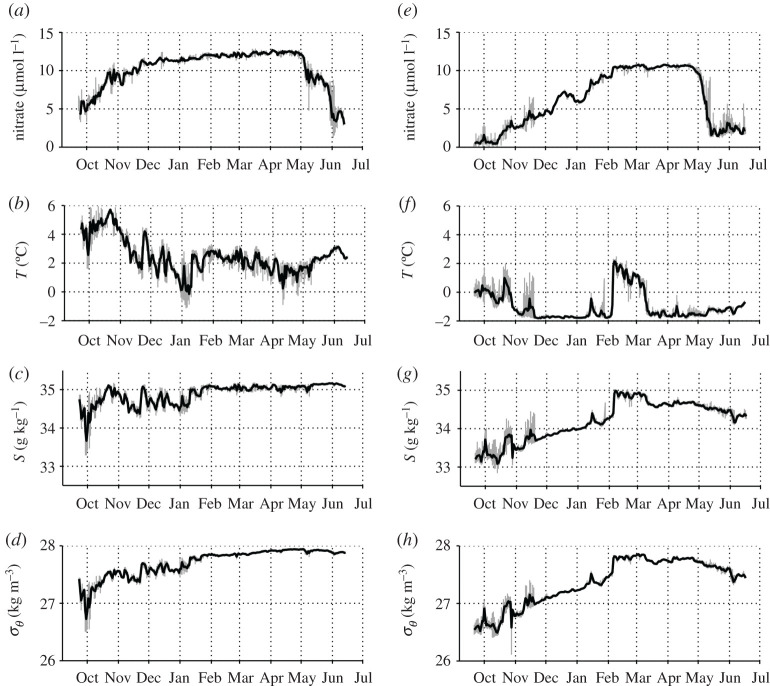
Time-series data from the uppermost instrument packages of both moorings at 21 m depth, September 2017 to June 2018. Shown are (*a*) nitrate concentration, (*b*) conservative temperature, (*c*) absolute salinity and (*d*) potential density (*σ_θ_*) at the western mooring and (*e*–*h*) eastern mooring. 2-hourly data are shown in grey, daily mean values in black.

Near-surface (21 m) nitrate concentration was lower than 1 µmol l^−1^ at the eastern mooring in late summer 2017 (figure 4), which is sufficiently low to limit phytoplankton growth [71,72]. Nitrate was replenished slowly over autumn and winter, and did not reach seasonal maximum values (greater than 10 µmol l^−1^) until February. In October and November, nitrate concentration increased while temperature, salinity and density were variable but showed an overall cooling and increases in salinity and density. In December and January, nitrate replenishment continued, concurrent with a steady increase in salinity and density and low temperatures typical of Arctic winter. The significant shift in water mass properties observed from early February to early March, which brought subsurface waters towards the surface, impacted strongly on near-surface temperature and salinity, but the signal in nitrate concentration was small. Maximum nitrate values (10.60 ± 0.08 µmol l^−1^) were observed between late March and late April, when salinity was high (around 34.7 g kg^−1^) and temperature was low (around −1.6°C). Temperature increased slightly and salinity decreased slightly into mid-summer 2018, driving a reduction in density from late April. By contrast, nitrate concentration was drawn down rapidly in early May from 9.31 µmol l^−1^ on 3 May to 1.60 µmol l^−1^ on 14 May at a drawdown rate of 0.67 ± 0.03 µmol l^−1 ^d^−1^. Nitrate concentrations remained low through summer 2018, with some variability most likely related to changes in nitracline depth around the SUNA. Nitrate concentrations of samples from the CTD profile taken on mooring recovery (electronic supplementary material, figure S2) from around the SUNA depth (±7 m) were 0.87 ± 0.49 µmol l^−1^, suggesting that SUNA measurements may be slightly high, by approximately 1 µmol l^−1^. However, the proximity of the upper nitracline to the SUNA depth means that minor (7 m) vertical displacement of water and associated gradients could have accounted for these differences and caused the variability observed in late May and early June. At the time of recovery, nitrate concentrations were limiting (less than or equal to 0.5 µmol l^−1^) in all samples collected above 20 m (electronic supplementary material, figure S2).

### (e) Seasonal variability in upper ocean nitrate concentration, temperature, salinity and density at the western mooring

Near-surface nitrate concentration was around 5 µmol l^−1^ at the western mooring in late September 2017 (figure 4), which is not sufficiently low to limit uptake by phytoplankton. These nitrate-replete conditions indicate either that nitrate did not reach limiting concentrations during summer or that nitrate concentrations had already started to recover from late-summer limitation when the mooring was deployed. Nitrate concentration increased rapidly during October alongside increases in temperature, salinity and density. Nitrate replenishment was slower during November, concurrent with decreases in temperature and salinity and a small decrease in density. Nitrate concentration was over 10 µmol l^−1^ by December and showed a sustained gradual increase over winter with maximum values of 12.20 ± 0.23 µmol l^−1^ between late January and late April. During this high-nitrate winter period, temperature was generally around 2°C with an overall downward trend, while salinity was high and stable (around 35.0 g kg^−1^), characteristic of well-mixed Atlantic-sourced water masses (table 2), and density was high and showed a small but measurable increase (27.84–27.94 kg m^−3^). From late April, temperature increased gradually while salinity remained high and stable and density remained high, but showed two small decreases in early May and early June. Nitrate was drawn down from 11.67 µmol l^−1^ on 4 May to 3.88 µmol l^−1^ on 1 June at an overall drawdown rate of 0.14 ± 0.01 µmol l^−1^ d^−1^. Within this period, there were two distinct drawdown pulses and an intervening period of relatively stable nitrate concentrations (8.90 ± 0.82 µmol l^−1^). Nitrate was drawn down from 11.67 µmol l^−1^ on 4 May to 8.90 µmol l^−1^ on 7 May at a rate of 0.91 ± 0.09 µmol l^−1 ^d^−1^. The second drawdown pulse reduced nitrate from 7.96 µmol l^−1^ on 28 May to 3.88 µmol l^−1^ on 1 June at a rate of 0.99 ± 0.11 µmol l^−1 ^d^−1^. Hence, nitrate drawdown occurred over a longer period than at the eastern mooring with a slower drawdown rate overall, although this rate was much faster during discreet periods. These drawdown pulses corresponded to rapid accumulations of phytoplankton biomass inferred from fluorescence measurements (electronic supplementary material, figure S3) and to the small decreases in upper ocean density. By the time of mooring recovery in mid-June, nitrate concentrations did not reach limiting levels at 21 m at the western mooring, and nitrate concentrations were over 2 µmol l^−1^ in all samples collected over the upper 20 m (electronic supplementary material, figure S2). This figure shows clearly the difference in nitrate concentrations, hydrographic parameters and chlorophyll between the two moorings, both over the full water column depth and around 21 m.

## 4. Discussion

### (a) The influence of sea ice and mixing on nutrient resupply during autumn and winter

Nitrate concentrations in near-surface waters over the northern Barents shelf were low at the end of summer 2017, then increased during autumn and winter to maximum values in late winter/early spring, and were drawn down rapidly by biological uptake during phytoplankton blooms in spring to low values that persisted through summer 2018. While this seasonal cycle was observed at both mooring locations, and is consistent with known seasonality in the region [14,24,25,28,51], there were important differences in the timing and magnitude of these processes driven primarily by differences in seasonal sea ice cover and its effect on upper ocean physics.

Under ice-free conditions at the western mooring, stratification breaks down quickly in early October leading to an increase in temperature and salinity in the near-surface waters and a rapid increase in nitrate concentration as nutrients are resupplied from nutrient-rich underlying waters (figure 4). Ice-free conditions and low stratification throughout winter maintain a high degree of vertical mixing, leading to high and gradually increasing near-surface nitrate concentration until spring. By contrast, at the eastern mooring, salinity-driven stratification is high through October and breaks down much more gradually (at least 3-fold slower than at the ice-free western mooring) from November to early February while sea ice is present. The sea ice cover probably restricts wind-driven vertical mixing, and the gradual decline in stratification leads to a delayed and much slower nutrient replenishment from underlying waters [51,73,74]. Although high winter nitrate concentrations were not reached until early February, these values persisted for almost three months prior to nitrate uptake in spring, such that slower autumn/winter resupply did not impact nutrient availability to phytoplankton at the onset of the spring bloom.

**Figure 5.**
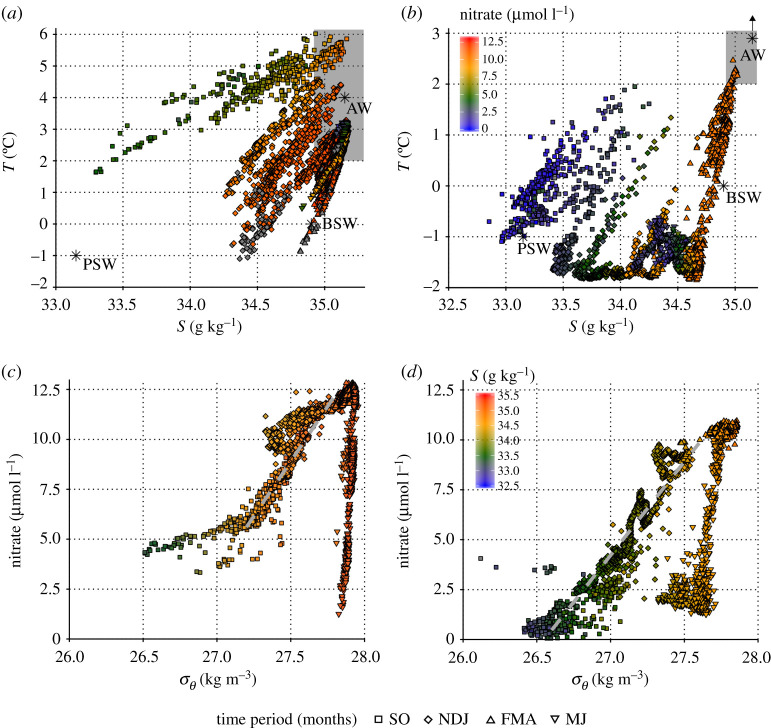
Temperature–salinity plots of near-surface (21 m) data for the (*a*) western and (*b*) eastern mooring. Data points are colour-coded by nitrate concentration and symbol depicts time period according to the major trends in the data. Grey-filled symbols indicate no nitrate data available. Single-point water mass definitions (table 2) are shown on each plot as well as the definition of AW in this region from Randelhoff *et al*. [52]. In (*b*), the single-point definition of AW is off-plot as indicated. Nitrate–density plots of near-surface data for the (*c*) western and (*d*) eastern mooring, with points colour-coded by salinity and symbols depicting time. Linear regressions (grey dashed lines) are for the periods of most pronounced increases in nitrate (5 October to 1 November at the western mooring, 30 September to 1 February at the eastern mooring).

The role of stratification and vertical mixing in regulating nitrate resupply is emphasized by the relationship between nitrate and density at each mooring, with similar slopes (10 ± 1 mmol kg^−1^) during the period of most pronounced increases in nitrate, but this period lasting much longer at the eastern mooring (figure 5). These plots show that changes in density had a much greater influence on nitrate concentrations during autumn-winter resupply than spring-summer drawdown, highlighting the importance of physical and biological drivers of nitrate dynamics operating over the seasonal cycle. A key difference between the two locations was that the seasonal maximum nitrate concentrations observed prior to springtime drawdown were higher (12.20 ± 0.23 µmol l^−1^) under ice-free conditions at the western mooring than at the ice-influenced eastern mooring (10.60 ± 0.08 µmol l^−1^), due to lower stratification and greater mixing in autumn, winter and early spring. This suggests that sea ice cover, and its influence on water column stratification, may reduce the initial pool of nitrate available to phytoplankton by restricting vertical nitrate supply, with the potential to reduce total primary production. A similar study conducted to the northeast of our eastern mooring in 2012–2013 [51] observed sea ice concentrations greater than 50% from February to June, and measured a similar seasonal nitrate maximum (10 µmol l^−1^) to our eastern mooring. Alongside evidence of smaller nitrate fluxes under ice than in open water in this region [73], this further supports our argument for sea ice cover limiting nutrient availability to spring phytoplankton blooms, and suggests that seasonal nitrate maxima of approximately 10 µmol l^−1^ may be broadly applicable to ice-influenced waters in this region.

### (b) Water masses, tidal processes and ocean currents as key regulators of nutrient availability

Near-surface nitrate concentrations are strongly linked to the nutrient content of different water masses and the structure and preponderance of these water masses throughout the water column. Temperature-salinity plots (figure 5) emphasize the difference in near-surface water mass properties between the two moorings, with warm, saline and nutrient-rich AW being much more prevalent at the western mooring from winter into summer, and a more minor influence of BSW, leading to higher nitrate concentrations overall. By contrast, near-surface properties at the eastern mooring were influenced by both AW and BSW, but PSW with lower nitrate concentrations dominated the upper layers for the majority of the study. This was particularly the case when sea ice was present, highlighting a strong influence of ice cover on water mass structure.

The large wind stress peak in early February and the prolonged ice-free period that this peak instigated caused a complete breakdown in stratification and entrained warmer, more saline and higher-nitrate subsurface waters dominated by AW and BSW towards the surface (figure 2). This is consistent with recent work in this region documenting wind-driven mixing layers as deep as 60 m [74]. In addition, currently unpublished data collected in spring and summer 2018 in the vicinity of the moorings suggest that the maximum wind stress observed in early February (0.87 N m^−2^; wind speed of 20 m s^−1^) can lead to mixing depths of up to 50 m (Rodgers, personal communication 2020). Peak wind stress and possible accompanying changes in wind direction (not examined here) may also have caused advection of water masses, as well as sea ice, which could have contributed to the observed signals. While the observed stratification breakdown only resulted in a small nitrate signal, high winter values (greater than 10 µmol l^−1^) were probably reached slightly earlier than if PSW had dominated throughout. The entrainment of deeper water masses did not make a major difference to nitrate availability when it occurred in winter, because near-surface concentrations were already high due to erosion of the nitracline by autumn/winter vertical mixing, and uptake by phytoplankton was negligible. However, if a similar event occurred when the nitracline was stronger and phytoplankton were growing, and wind stress was sufficient to overcome the increased spring/summer stratification, this wind/ice-driven mixing/entrainment mechanism could enhance nitrate availability to primary producers in surface waters. These observations implicate a key role for wind and sea ice forcing of water mass structure, stratification and potentially nutrient availability, with the importance of these forcings and their consequences dependent on the time of year and biogeochemical processes at work [25,37,39,74]. These ice-free periods and areas within the variable ice cover are likely to become more common in the Barents shelf seasonal ice zone as declines in winter sea ice concentration continue and ice retreat occurs progressively earlier in spring, such that the processes documented here would become more important until this region is ice-free year-round, potentially from the 2050s [7,8,54].

High-resolution plots of unsmoothed water mass percentage data reveal a strong tidal component in water mass structure at the eastern mooring with a periodicity of approximately 12 h (electronic supplementary material, figure S4a). Tidally-induced vertical movement is known to increase shear between layers, providing a mechanism for enhanced nutrient fluxes into surface waters [75,76]. While the nutrient-rich AW remains below the pycnocline throughout, the continuous tidal activity enhances shear and thus vertical nutrient fluxes, thereby contributing to the gradual nutrient replenishment of surface waters between late September and early February, particularly during the ice-covered months when wind forcing is reduced. At the western mooring (electronic supplementary material, figure S4b), the role of tidally-induced shear in vertical nutrient fluxes is reduced because the upper water column is less stratified and dominated by nutrient-rich AW throughout. In agreement with previous findings from the Barents Sea [37,77], we propose that vertical nutrient resupply over the northern Barents shelf is driven by both wind and tidal forcing, with tidal forcing having a greater influence at the eastern mooring where wind-driven mixing is less intense. These forcings also operate on different timescales, with tidal forcing providing a continuous yet lower rate of mixing and wind events being episodic yet leading to higher rates of mixing, especially if they initiate convective overturning [52,77].

Variability in the strength and position of the Svalbard branch of the AW boundary current, which advects AW over the mooring sites, influences observed near-surface and subsurface water properties substantially, and is known to dominate over cross-shelf-slope transports in this region [51]. The greater influence of this current and more Atlantic-like conditions at the western mooring contribute to its higher temperature, salinity and nitrate concentrations than at the eastern mooring, and potentially drive the observed differences in sea ice dynamics and upper ocean stratification [52,54,55,78]. This influence also means that the advective component of observed variability in nitrate concentrations and other parameters is likely to be more important at the western mooring, such that these results integrate processes occurring over a larger upstream area than those from the eastern mooring. However, previous work has shown that a similar seasonal cycle of nitrate drawdown and resupply in Fram Strait and northwest of Svalbard leads to similar vertical nitrate gradients upstream of the moorings [51,52], such that horizontal advection makes a minor contribution to the observed seasonal patterns driven primarily by vertical nitrate fluxes and biological uptake.

### (c) Biological nitrate drawdown and phytoplankton bloom dynamics

#### (i) Initiation of nitrate drawdown and phytoplankton blooms

Pronounced nitrate drawdown driven by biological uptake by phytoplankton is observed at both mooring locations in spring, with the onset of nitrate drawdown occurring in early May. The near-synchronous initiation of nitrate drawdown under ice-influenced and ice-free conditions at the same latitude invokes the availability of light from the sun as the primary control on the timing of the onset of nutrient uptake and, by inference, phytoplankton growth. The control by light availability of phytoplankton growth onset is consistent with non-limiting nutrient concentrations early in the spring/summer growing season, and suggests that stratification at both moorings was sufficient to retain cells in the well-lit upper ocean [79,80], despite fundamental differences in sea ice conditions and their effect on the upper water column. The first 4–5 days of nitrate drawdown at the eastern mooring occurred while satellite-derived ice concentration was greater than 75%, suggesting that the initial development of the spring phytoplankton bloom occurred under sea ice, as has been observed elsewhere in the Arctic [81–83], or at least in an area strongly influenced by ice. This requires sufficient light to have reached the surface waters to support phytoplankton growth at that time, either through the ice pack or into open water areas within a patchy ice cover [51,84]. These under-ice or ice-edge phytoplankton blooms could have been seeded by sympagic (ice-associated) algae [85,86] and/or developed from senescent cells or resting spores in the water column [10,87,88], but the relative importance of these seeding mechanisms is unknown for this study. Our findings show that sea ice cover is not the most important control on the timing of onset of biological nitrate drawdown, but we go on to show the important control of ice dynamics on the rate and duration of this nutrient drawdown by phytoplankton blooms in this region.

#### (ii) Nitrate drawdown under ice-influenced versus ice-free conditions

The observed patterns of springtime nitrate drawdown reflect biological uptake by phytoplankton, compensated to some degree by nitrate resupply primarily through vertical mixing. Horizontal patchiness and advection of surface properties could have caused small-scale variability superimposed on the larger seasonal patterns [51]. At the eastern mooring, ice-influenced conditions and a more stratified upper ocean facilitated rapid and monotonic nitrate drawdown from high winter values to low concentrations over twelve days in early May. This is partly because more stratified conditions provided a shallow well-lit mixed layer favourable for phytoplankton growth and efficient nitrate uptake (e.g. [73,79,80]), and partly because greater stratification reduced nitrate resupply by vertical mixing during the growing season, leading to faster depletion of the nutrient pool (e.g. [33,73,89]). Under ice-free conditions at the western mooring, the longer period of nitrate drawdown, with an overall drawdown rate four times slower than at the eastern mooring, was likely caused by slower phytoplankton growth. Less stratified conditions would have mixed phytoplankton cells over a larger depth interval, exposing them to more variable light intensities and lowering their growth rates. The two distinct drawdown pulses in early and late May coincided with short-lived increases in stratification and rapid accumulation of phytoplankton biomass (electronic supplementary material, figure S3), indicating a bimodal peak in primary production facilitated by increased mixed layer stability. The intervening period of relatively stable nitrate concentrations and a reduction in phytoplankton biomass coincided with enhanced wind stress (greater than 0.25 N m^−2^) in mid-May, suggesting that increased wind-mixing dispersed phytoplankton over greater depths and likely enhanced nutrient resupply from underlying waters. Variability in the influence of the AW boundary current (§4b) and advection of a higher-nitrate lower-biomass patch through the mooring location could also explain the apparent slow-down of nitrate drawdown, at least in part. Slower nitrate drawdown overall at the western mooring and potentially greater nitrate resupply during the growing season are likely to extend the length of the phytoplankton growing season compared to the ice-influenced mooring, with short-lived periods of rapid drawdown contributing substantially to total primary production. However, a reliable comparison of total nitrate uptake, and therefore primary production, between the rapid monotonic nitrate drawdown observed under ice-influenced conditions and the slower but longer-lasting drawdown under ice-free conditions is not possible with this dataset.

#### (iii) Nitrate as the limiting nutrient for phytoplankton growth

Important differences in the degree of nitrate drawdown and the potential for nitrate limitation of phytoplankton growth were also observed between the moorings. Bloom-forming Arctic phytoplankton become nitrate-limited at concentrations of 0.5 to 1 µmol l^−1^ [71,72]. At the western mooring, nitrate concentrations were well above these limits in late-summer 2017 and summer 2018, likely because of a greater degree of mixing linked to the greater AW influence.

At the eastern mooring in summer 2018, low nitrate concentrations measured by the SUNA and limiting concentrations in the upper 20 m strongly suggest nitrate limitation of surface phytoplankton blooms. This is consistent with lower chlorophyll concentrations over the upper 40 m than at the western mooring (electronic supplementary material, figure S2). Non-limiting nitrate concentrations below the SUNA depth had not led to formation of a subsurface chlorophyll maximum by mid-June, but these features may become more important in maintaining productivity, albeit at lower rates, later in the summer as nitrate limitation intensifies in surface waters and the nitracline continues to deepen [21,90]. Near-surface nitrate concentrations lower than 1 µmol l^−1^ measured by moored nitrate sensors in September 2017 (this study), September 2012 and July 2013 [51] also indicate nitrate limitation in this region, and add to the body of evidence showing that summertime nitrate limitation is common in the northern Barents shelf seasonal ice zone [52,73,91].

The extent to which uptake of ammonium and other regenerated nitrogen forms can sustain phytoplankton blooms once nitrate becomes limiting cannot be assessed with the current dataset. However, ammonium-based production is likely to occur once ammonium is made available by organic matter recycling, because such reduced compounds are assimilated more easily than nitrate, and may make an important contribution to total primary productivity [24,91–94]. The supply and uptake of inorganic and organic nitrogen over the Barents shelf is the subject of ongoing work. Our observations of limiting nitrate concentrations in surface waters at the eastern mooring and non-limiting nitrate concentrations at the western mooring in summers 2017 and 2018 show that nitrate availability to phytoplankton is reduced under ice-influenced conditions compared to ice-free waters. This is likely to be caused by the influence of sea ice on upper ocean stratification and its impact on vertical nutrient supply (§§4a and 4b) and nitrate uptake efficiency (§4c(ii)), in agreement with other studies in this region [14,51,73].

### (d) Synthesis and implications under ongoing change

The strong seasonal cycle of near-surface nitrate concentration and physical oceanographic parameters observed under ice-influenced and ice-free conditions over the northern Barents shelf has important implications for nutrient supply and uptake in the context of ongoing warming, ice losses and Atlantification. The timing, rate and duration of nitrate drawdown by phytoplankton spring blooms support previous findings that primary production in this Arctic shelf region is co-limited by light and nutrient availability [10,22,24,28]. The near-synchronous initiation of nitrate drawdown under nutrient-replete conditions at both mooring locations strongly suggests that the timing of phytoplankton growth onset is regulated by increasing light levels as the sun returns to high northern latitudes, whether or not sea ice is present and regardless of observed differences in water column structure and properties. Nutrient availability becomes a more important limiting factor later in the growing season, with the duration of phytoplankton blooms being regulated by nutrient supply preceding and during the growing season. The availability of both light and nutrients will thus be important controls on primary production as climate change proceeds [14,17,21,22,33].

While sea ice and upper ocean conditions did not appear to affect the timing of phytoplankton growth onset, they did influence nutrient availability and drawdown over the growing season. We show that nitrate availability was higher under ice-free conditions, both prior to and after the period of pronounced drawdown in spring and early summer. Nitrate drawdown was also sustained for longer, potentially with greater nitrate resupply during that period. However, the rate of drawdown was slower under ice-free conditions, and we ascribe this to reduced phytoplankton growth rates as cells were mixed to greater depths with more variable light levels due to weaker stratification than at the ice-influenced mooring.

The stronger influence of the AW boundary current and more Atlantic-like conditions at the western mooring play an important role in driving the ice-free conditions and their impact on nutrient supply and uptake. Previous work has shown that more Atlantic-like conditions in this region lead to later spring blooms due to later stratification [52]. We did not observe a similar difference in the timing of onset of nitrate drawdown and we suggest that this is because stratification was sufficient to permit phytoplankton growth at both moorings by early May (§4c(i)). Nevertheless, our observations of slower nitrate drawdown under more Atlantic-like conditions do support more sustained biological uptake, and agree with blooms persisting later into the summer and likely peaking later than under more Arctic-like conditions in the seasonal ice zone.

In the context of ongoing Arctic climate change and Atlantification of the Barents shelf [7,8,54–56,78,95,96], it is reasonable to expect the conditions that we observed at the western mooring to become more widespread in the coming decades and to replace the conditions observed at the eastern mooring across large parts of the Barents shelf seasonal ice zone. It has been hypothesized that increased advection and influence of AW in this region is alleviating both light and nutrient limitation of phytoplankton growth [22,52], compounding the documented increases in primary production driven by ongoing increases in light availability as ice-influenced regions transition to ice-free conditions and ice-free periods lengthen [13,15]. Our findings suggest that ongoing warming, ice losses and Atlantification could increase nutrient availability and the duration of seasonal drawdown in the surface ocean. The extent to which this increased nutrient availability and longer drawdown periods will lead to increases in total nitrate uptake and therefore primary production will depend on changes in upper ocean stratification also occurring as a result of ice losses and Atlantification, and their effect on light availability to phytoplankton. Changes in phytoplankton species composition are also likely to be influenced by changing sea ice dynamics, water column stratification and nutrient availability, and need to be considered in assessments and projections of primary production, food web dynamics and ecosystem carbon storage [10,18,21,62,97]. If the increase in nutrient supply from ice-influenced to ice-free conditions documented here does lead to increases in primary production over the northern Barents shelf as warming, ice losses and Atlantification proceed, biological carbon uptake and export would also increase, with consequences for air-sea CO_2_ exchange and ocean-climate feedbacks in this globally important and climatically sensitive region [14,21,89].

## Supplementary Material

Supplementary figures

## Supplementary Material

Data

## Data accessibility

Data presented in this study are publicly available through the British Oceanographic Data Centre (BODC) as well as being provided as part of the electronic supplementary material for this article. Wind and sea ice data are not included, because they are publicly available as detailed in the article text and reference list. Stratification index, mixed layer depth and water mass percentages are also not included as they are derived parameters as explained in the text.
