# Supplementary material for: Nitrate supply and uptake in the Atlantic Arctic sea ice zone: seasonal cycle, mechanisms and drivers
Source: Philos Trans A Math Phys Eng Sci. 2020 Aug 31;378(2181):20190361. doi: 10.1098/rsta.2019.0361 (PMC7481658; doi:10.1098/rsta.2019.0361)
Supplement: Supplementary figures [file rsta20190361supp1.pdf]

Nitrate supply and uptake in the Atlantic Arctic sea ice zone: seasonal cycle, mechanisms and drivers

Sian F. Henley, Marie Porter, Laura Hobbs, Judith Braun, Robin Guillaume-Castel, Emily J. Venables, Estelle Dumont, Finlo Cottier

Philosophical Transactions of the Royal Society A. DOI: 10.1098/rsta.2019.0361

Supplementary information

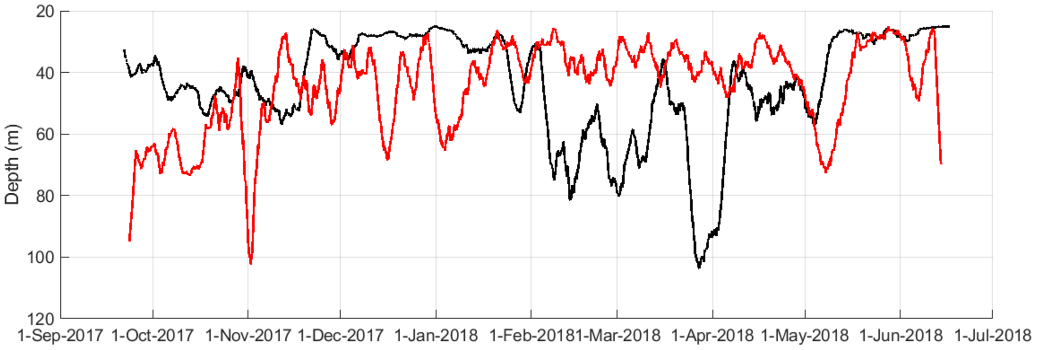

Figure S1. Mixed layer depth represented by the depth of the maximum buoyancy frequency (N^2) at the eastern (black line) and western (red line) moorings over the duration of the deployments.

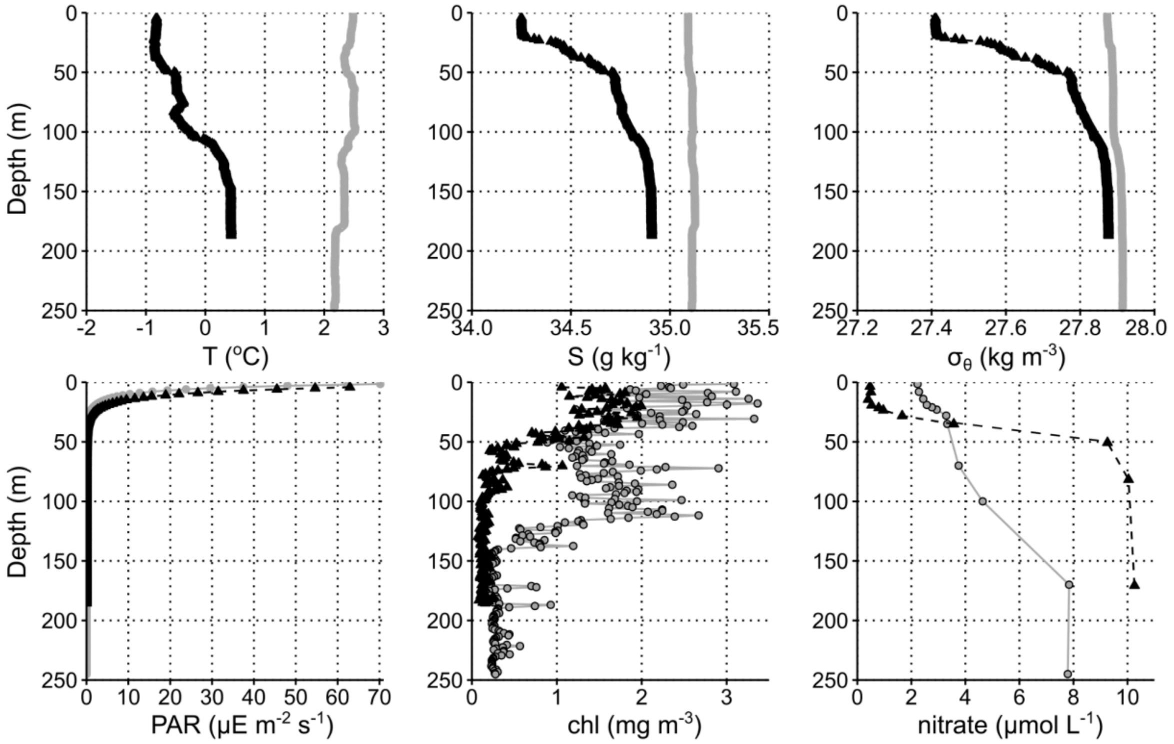

Figure S2. CTD profiles taken on mooring recovery in mid-June 2018 at the eastern (black) and western (grey) moorings. Shown are conservative temperature, absolute salinity, potential density (σ_θ), photosynthetically active radiation, fluorescence-derived chlorophyll and nitrate concentration from bottle samples. This figure shows clearly the difference in nitrate concentrations, hydrographic parameters and chlorophyll between the two moorings, both over the full water column depth and around 21 m.

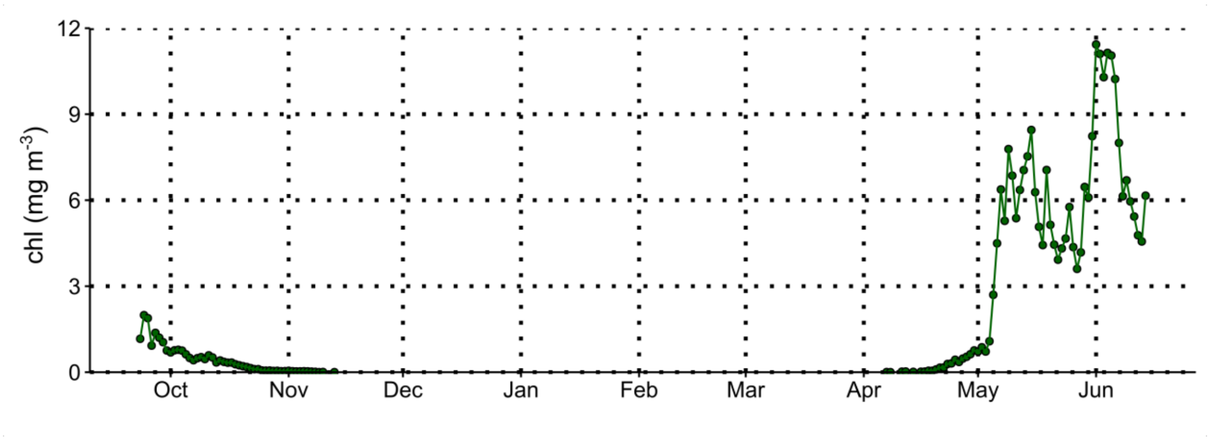

Figure S3. Daily mean fluorescence-derived chlorophyll concentration at 21 m depth at the western mooring. Comparable data are not available for the eastern mooring because the SBE16 failed.

a) Eastern mooring water masses and density

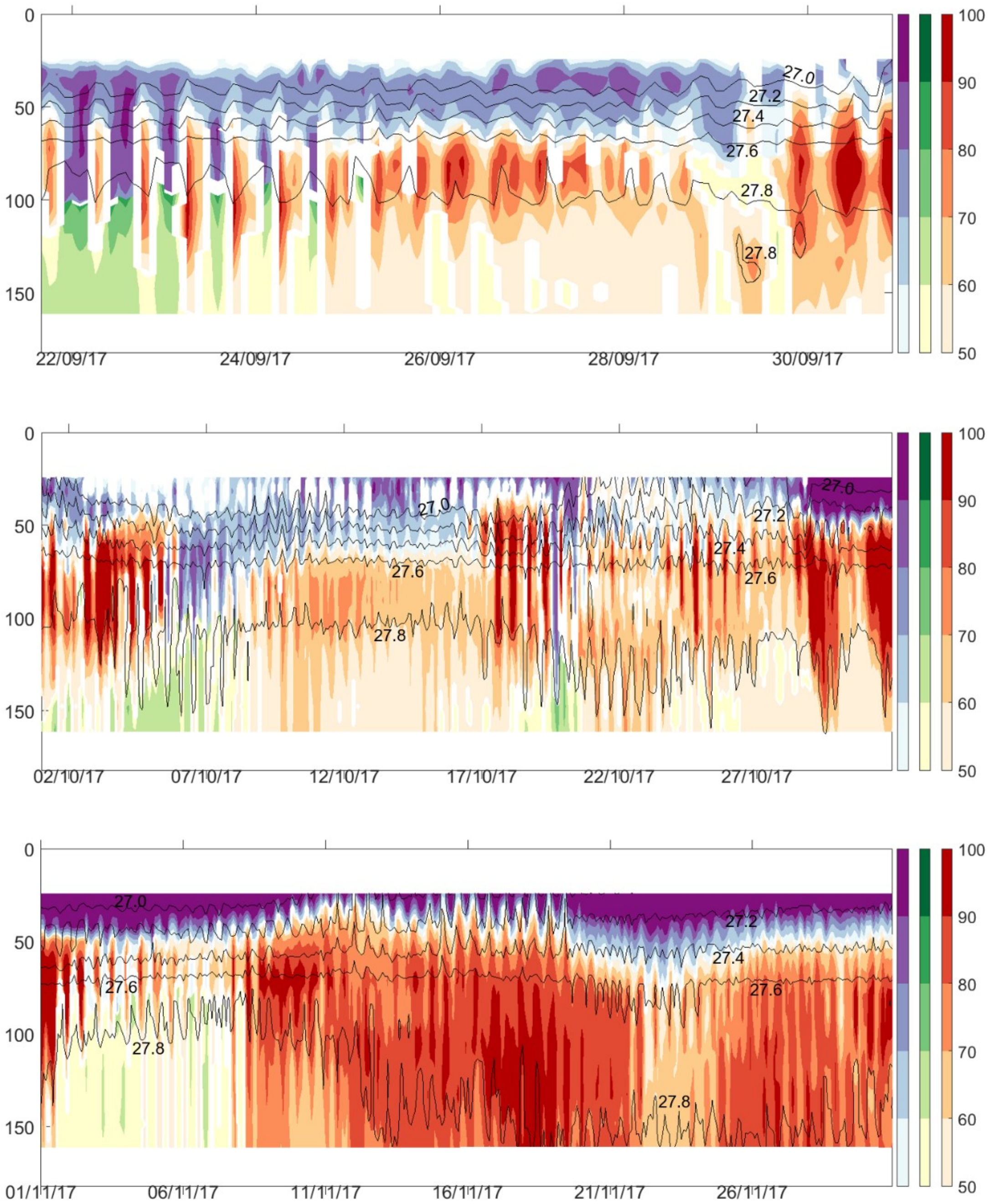

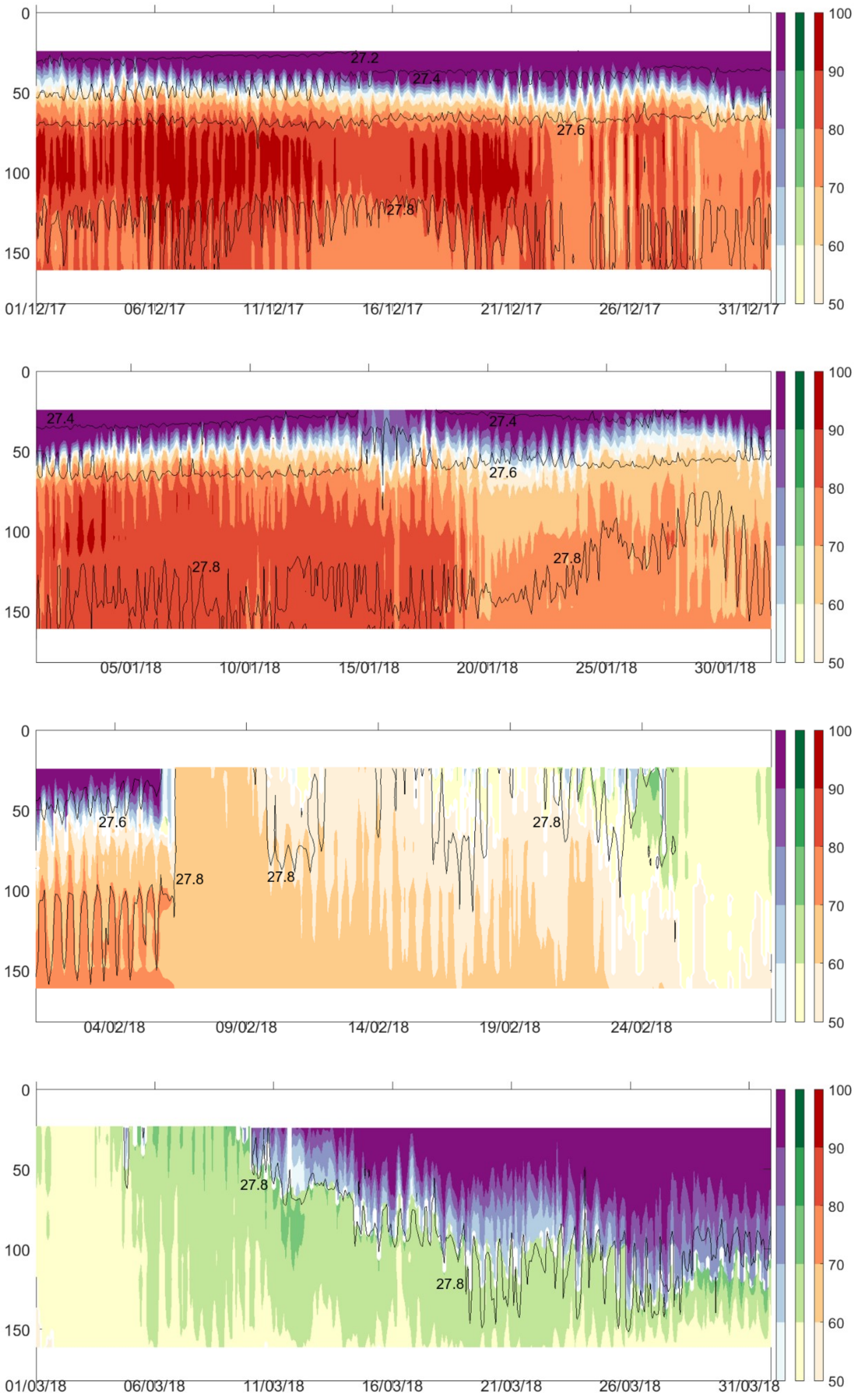
0
50
100
150
27.2
27.4
27.6
27.8
01/12/17
06/12/17
11/12/17
16/12/17
21/12/17
26/12/17
31/12/17
100
90
80
70
60
50
27.4
27.6
27.8
05/01/18
10/01/18
15/01/18
20/01/18
25/01/18
30/01/18
27.6
27.8
04/02/18
09/02/18
14/02/18
19/02/18
24/02/18
27.8
01/03/18
06/03/18
11/03/18
16/03/18
21/03/18
26/03/18
31/03/18

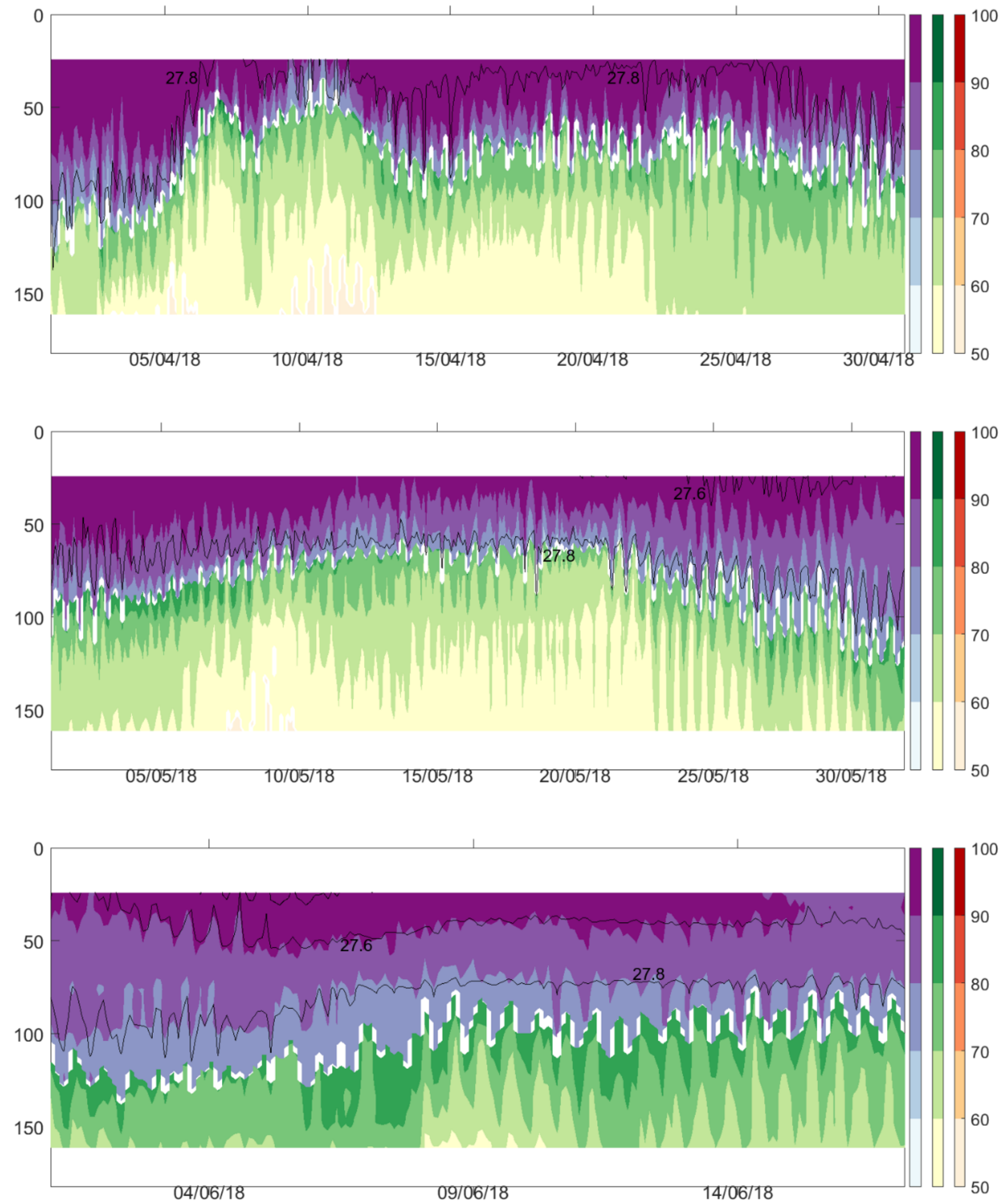

27.8
27.8
05/04/18
10/04/18
15/04/18
20/04/18
25/04/18
30/04/18
27.6
27.8
05/05/18
10/05/18
15/05/18
20/05/18
25/05/18
30/05/18
27.6
27.8
04/06/18
09/06/18
14/06/18

b) Western mooring water masses and density

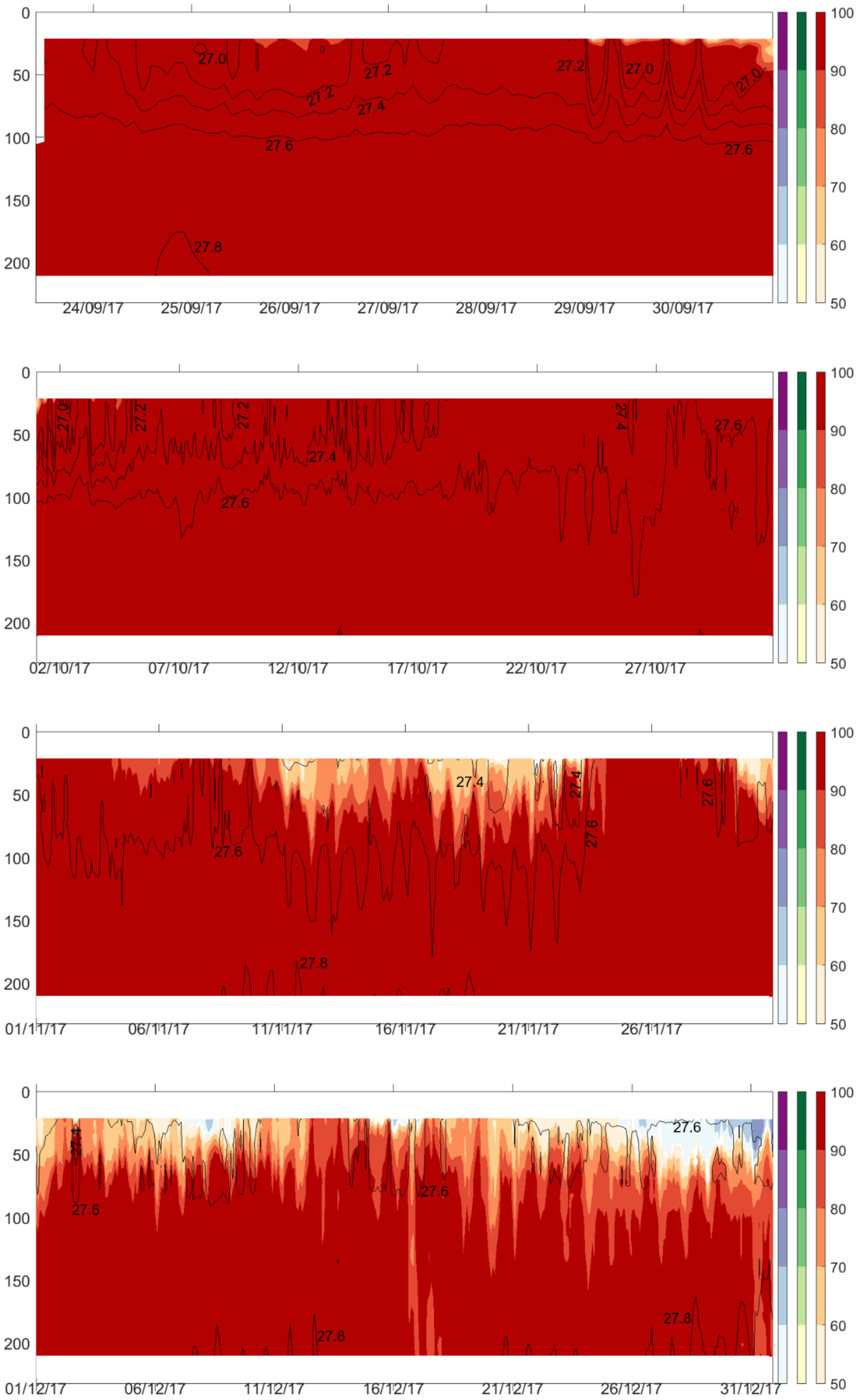

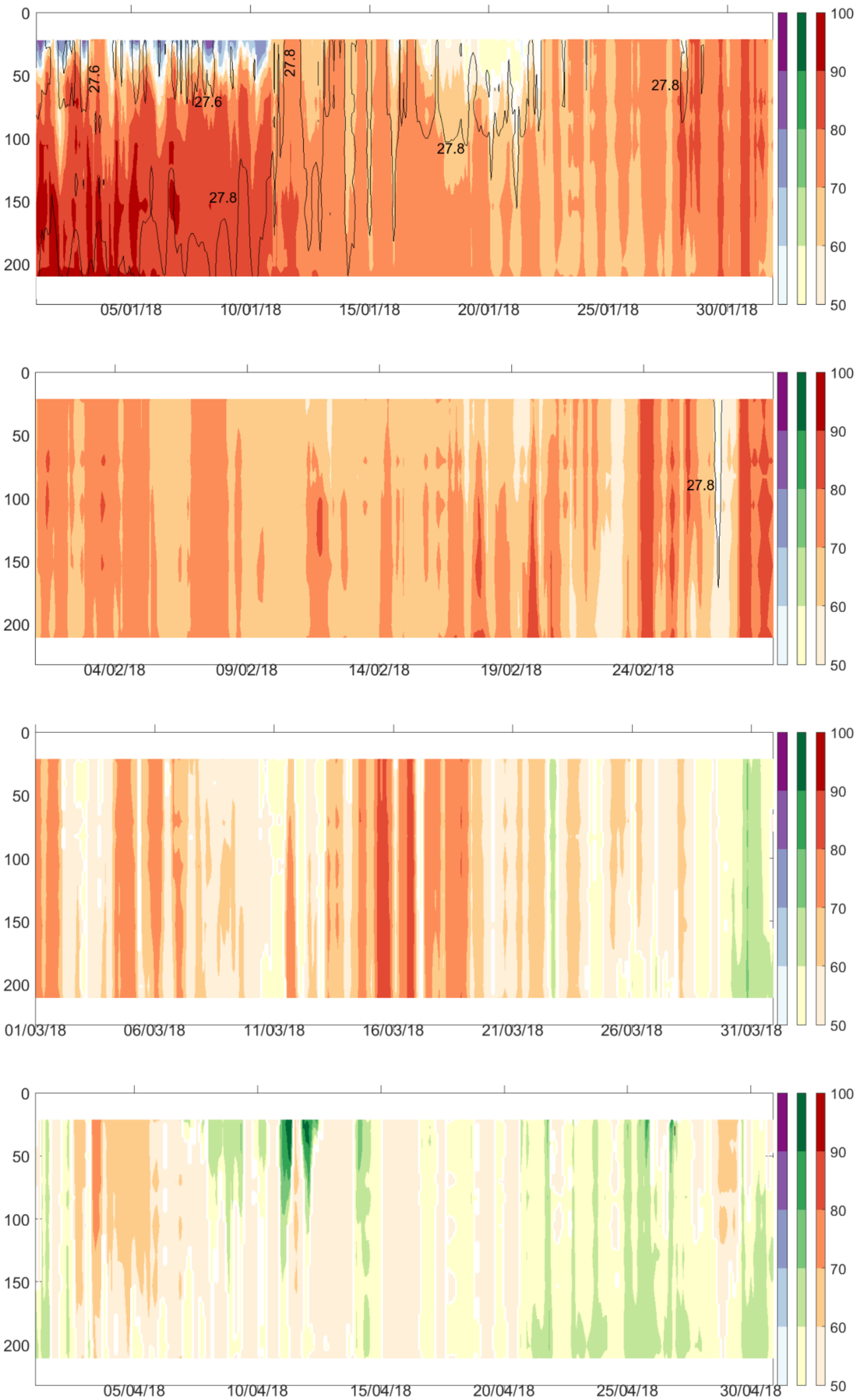
0
50
100
150
200
27.6
27.6
27.8
27.8
27.8
27.8
05/01/18
10/01/18
15/01/18
20/01/18
25/01/18
30/01/18
100
90
80
70
60
50
27.8
04/02/18
09/02/18
14/02/18
19/02/18
24/02/18
01/03/18
06/03/18
11/03/18
16/03/18
21/03/18
26/03/18
31/03/18
05/04/18
10/04/18
15/04/18
20/04/18
25/04/18
30/04/18

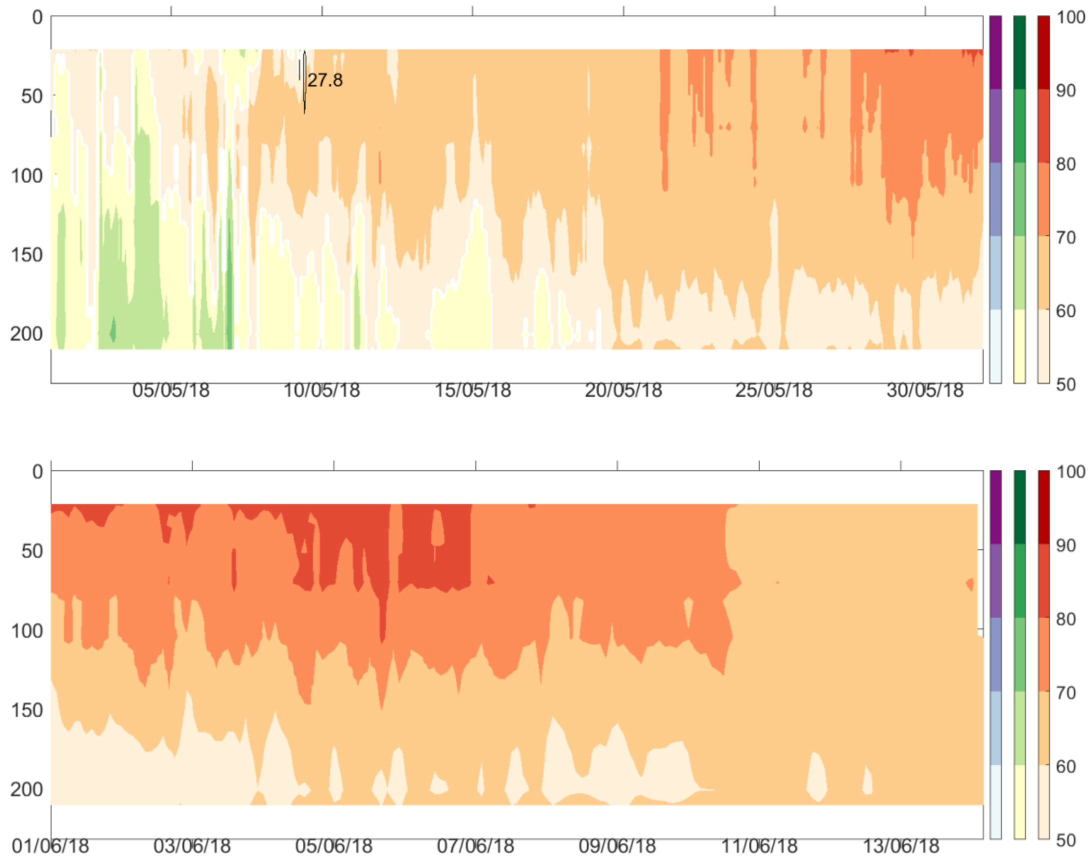

Figure S4. High-resolution unsmoothed water mass percentages for September 2017 to June 2018, calculated from temperature and salinity, with isopycnals at 0.2 kg m^{-3} intervals overlain. Polar Surface Water (PSW) = purple, Barents Sea Water (BSW) = green, Atlantic Water (AW) = red. (a) monthly fields of the upper 170 m for the eastern mooring, (b) monthly fields of the upper 200 m for the western mooring. As well as tidal variability of isopycnal depths, alternation of AW and BSW dominance in subsurface waters is observed at both mooring locations. We suggest that an onshore-offshore gradient between AW and BSW is being advected back and forth across the shelf, driving this alternation. This is most evident during discreet periods between late September and mid-November and in mid-late February at the eastern mooring, and from late March until early May at the western mooring.
